# Evaluation of patient‐reported outcome measures for on‐demand treatment of hereditary angioedema attacks and design of KONFIDENT, a phase 3 trial of sebetralstat

**DOI:** 10.1002/clt2.12288

**Published:** 2023-09-04

**Authors:** Danny M. Cohn, Emel Aygören‐Pürsün, Jonathan A. Bernstein, Henriette Farkas, William R. Lumry, Marcus Maurer, Andrea Zanichelli, Matthew Iverson, James Hao, Michael D. Smith, Christopher M. Yea, Paul K. Audhya, Marc A. Riedl

**Affiliations:** ^1^ Department of Vascular Medicine, Amsterdam Cardiovascular Sciences Amsterdam UMC University of Amsterdam Amsterdam The Netherlands; ^2^ Department for Children and Adolescents University Hospital Frankfurt Frankfurt Germany; ^3^ Division of Rheumatology, Allergy and Immunology Department of Internal Medicine University of Cincinnati College of Medicine Cincinnati Ohio USA; ^4^ Hungarian Angioedema Center of Reference and Excellence Department of Internal Medicine and Haematology Semmelweis University Budapest Hungary; ^5^ AARA Research Center Dallas Texas USA; ^6^ Institute of Allergology Charité ‐ Universitätsmedizin Berlin Corporate Member of Freie Universität Berlin and Humboldt‐Universität zu Berlin Berlin Germany; ^7^ Allergology and Immunology Fraunhofer Institute for Translational Medicine and Pharmacology ITMP Berlin Germany; ^8^ Operative Unit of Medicine Angioedema Center IRCCS Policlinico San Donato Milan Italy; ^9^ Department of Biomedical Sciences for Health University of Milan Milan Italy; ^10^ KalVista Pharmaceuticals Cambridge Massachusetts USA; ^11^ KalVista Pharmaceuticals Salisbury UK; ^12^ Division of Rheumatology, Allergy & Immunology University of California San Diego San Diego California USA

**Keywords:** HAE, hereditary angioedema, KONFIDENT, Patient Global Impression of Change, sebetralstat, study design

## Abstract

**Background:**

Hereditary angioedema (HAE) with C1‐inhibitor deficiency (HAE‐C1‐INH) is characterized by recurrent, debilitating episodes of swelling. Sebetralstat, an investigational oral plasma kallikrein inhibitor, demonstrated promising efficacy for on‐demand treatment of HAE‐C1‐INH in a phase 2 trial. We describe the multipronged approach informing the design of KONFIDENT, a phase 3 randomized, placebo‐controlled, three‐way crossover trial evaluating the efficacy and safety of sebetralstat in patients aged ≥12 years with HAE‐C1‐INH.

**Methods:**

To determine an optimal endpoint to measure the beginning of symptom relief in KONFIDENT, we engaged patients with HAE on clinical outcome measures and subsequently conducted analyses of phase 2 outcomes. Sample size was determined via a simulation‐based approach using phase 2 data.

**Results:**

Patient interviews revealed a strong preference (71%) for the Patient Global Impression of Change (PGI‐C) over other measures and indicated a rating of “A Little Better” as a clinically meaningful milestone. In phase 2, a rating of “A Little Better” demonstrated agreement with attack severity improvement and resolution on the Patient Global Impression of Severity and had better sensitivity than “Better.” Simulations indicated that 84 patients completing treatment would ensure at least 90% power for assessing the primary endpoint of time to beginning of symptom relief defined as a PGI‐C rating of at least “A Little Better” for two time points in a row.

**Conclusions:**

Patient feedback and phase 2 data support PGI‐C as the primary outcome measure in the phase 3 KONFIDENT trial evaluating sebetralstat, which has the potential to be the first oral on‐demand treatment for HAE‐C1‐INH attacks.

## 1 INTRODUCTION

Hereditary angioedema (HAE) is a rare genetic disease characterized by unpredictable, recurrent episodes of mucosal swelling (referred to as HAE attacks).^1^, ^2^ The prevalence of HAE is estimated to be approximately 1 in 50,000 people worldwide.^3^ HAE is most commonly caused by C1‐inhibitor deficiency, resulting from either reduced expression (type I HAE) or reduced functional activity (type II HAE).^1^ C1‐inhibitor deficiency facilitates excessive formation of plasma kallikrein, cleavage of high‐molecular‐weight kininogen, and generation of the vasodilator bradykinin.^1^ This overactivity of the kallikrein‐kinin system results in increased vascular permeability and angioedema.^1^ HAE attacks can affect any part of the body, including the hands, feet, face, gastrointestinal tract, genitals, and upper airways; attacks may involve more than one location simultaneously.^1^ They are often debilitating and may be life‐threatening when the upper airways are affected.^1^, ^2^

Currently approved treatments for HAE with C1‐inhibitor deficiency (HAE‐C1‐INH) include on‐demand therapies (plasma‐derived or recombinant C1‐inhibitor, the bradykinin B2 receptor antagonist icatibant, and the plasma kallikrein inhibitor ecallantide [US only]) and prophylactic treatments (plasma‐derived C1‐inhibitor, the anti‐plasma kallikrein antibody lanadelumab, and the plasma kallikrein inhibitor berotralstat).^4^, ^5^, ^6^, ^7^, ^8^, ^9^, ^10^, ^11^, ^12^ In general, the availability and utilization of prophylactic treatments varies widely across countries, and many patients continue to experience HAE attacks.^13^

**FIGURE 1:**
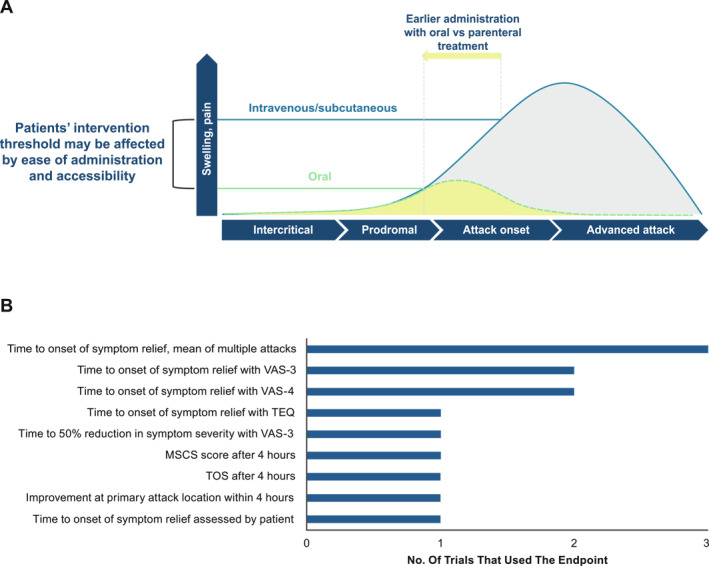
Hereditary Angioedema Attack Progression, Outcomes, and Study Endpoints. A) HAE attack progression and outcomes following earlier treatment^a,b^ and B) primary endpoints in 13 trials of HAE on‐demand therapy.^20^ HAE, hereditary angioedema; MSCS, mean symptom complex severity; TEQ, Treatment Effect Questionnaire; TOS, treatment outcome score; VAS, visual analog scale. ^a^Model based on Maurer et al. *PLoS One*, 2013.^21^ ^b^Figure adapted from Zanichelli, 2021.^22^

Treatment guidelines recommend that all patients with HAE should have access to effective on‐demand therapies for HAE attacks and that attacks should be treated as soon as possible^3^ as early treatment has been shown to reduce the severity and duration of an attack and improve quality of life.^3^, ^4^ However, all approved on‐demand treatment options must be administered parenterally (either subcutaneously or intravenously),^4^ which adds complexity to patient decision‐making and may contribute to undertreatment or delays in treatment. In real‐world observational studies, treatment delays were evident, with a median time to self‐administration after attack onset of 2 h for intravenous C1‐inhibitor and 1.5 h for subcutaneous icatibant.^14^, ^15^ A study has shown that treatment was delayed for >5 h in 26% of attacks that were treated with icatibant.^14^ Delayed treatment may be driven by a number of factors, including the time required to obtain and prepare medication, hesitation related to anticipated pain or other adverse effects associated with injection or infusion, and the need to find a discreet and hygienic location to administer treatment.^16^, ^17^, ^18^, ^19^ Patients may also delay the treatment of attacks that are not deemed to be of sufficient severity or those initially isolated to peripheral anatomic locations.^16^ There is an unmet need for an effective, oral, on‐demand treatment that would eliminate many of the complexities associated with parenteral therapies and enable patients to treat attacks as early as possible, increasing the likelihood of rapid symptom relief and minimizing attack duration and severity (Figure 1A).

**FIGURE 2:**
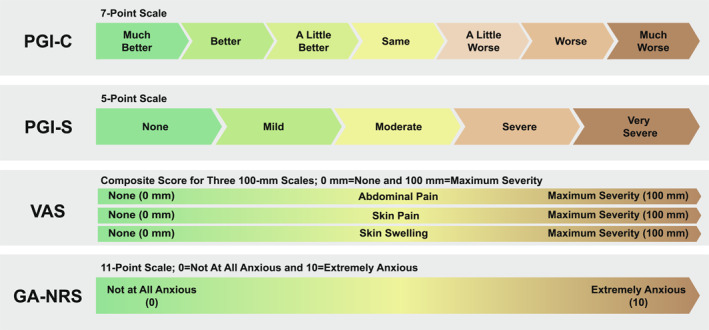
Efficacy Assessment Scales. GA‐NRS, General Anxiety–Numeric Rating Scale; PGI‐C, Patient Global Impression of Change; PGI‐S, Patient Global Impression of Severity; VAS, visual analog scale.

Because HAE attacks span a diversity of symptoms, anatomic locations, and levels of severity, assessment of response to therapy relies on patient‐reported outcome (PRO) measures.^20^ PRO measures are also practical for assessing symptom relief in HAE because attacks are most often self‐treated outside medical facilities. However, there are no standard PRO measures of acute response to therapy and HAE symptom relief, with various outcome measures and instruments used across trials^20^ (Figure 1B). In a double‐blind, phase 2, placebo‐controlled, two‐way crossover trial of sebetralstat (NCT04208412),^19^ an investigational oral plasma kallikrein inhibitor, efficacy was assessed by a number of measures: time to use of conventional on‐demand medication, Patient Global Impression of Change (PGI‐C), Patient Global Impression of Severity (PGI‐S), and a composite visual analog scale (VAS) comprising skin pain, abdominal pain, and skin swelling (Figure 2).

In order to design a suitable phase 3 clinical trial to confirm the efficacy and safety of sebetralstat, we aimed to identify an optimal efficacy measure to evaluate the response to on‐demand treatment of attacks in HAE‐C1‐INH. Here, we describe the multipronged approach that provided the rationale for the selection of time to beginning of symptom relief defined as a PGI‐C rating of at least “A Little Better” for two time points in a row within 12 h of the first study drug administration as the primary efficacy endpoint in the phase 3 sebetralstat trial (KONFIDENT, NCT05259917). Additionally, we describe the statistical model that informed the trial design of this randomized, double‐blind, placebo‐controlled, event‐driven, three‐way crossover trial.

## 2 METHODS

### 2.1 Overview of the phase 2 trial of sebetralstat

Details of the phase 2 trial of sebetralstat have been reported elsewhere.^19^ In brief, in part 2 of this trial, 53 patients aged ≥18 years with HAE‐C1‐INH treated two eligible (mild or moderate severity) HAE attacks with oral sebetralstat (600 mg) or placebo in a double‐blind, placebo‐controlled, crossover design. In addition to achieving the trial's primary endpoint, with sebetralstat significantly increasing time to use of conventional therapy within 12 h versus placebo (*p* = 0.001), sebetralstat resulted in earlier symptom relief than placebo, with a median time to symptom relief using the PGI‐C (defined as a rating of at least “A Little Better” for two consecutive time points within 12 h) of 1.6 h (95% CI: 1.5–3.0) with sebetralstat versus 9.0 h (95% CI: 4.0–17.2) with placebo (*p* < 0.0001) and median time to symptom relief using the composite VAS (defined as 50% reduction from baseline for three consecutive time points) of 6.0 h (95% CI: 3.0–9.0) with sebetralstat versus > 12 h (95% CI: not evaluable‐not evaluable) with placebo (*p* < 0.0001).^19^ Further, patients who received sebetralstat reported a lower proportion of attacks with symptom worsening on the PGI‐S (21% vs. 45%; *p* = 0.0045) within 12 h and a greater proportion of attacks that achieved resolution on the PGI‐S (53% vs. 26%; *p* = 0.012) within 24 h than those who received placebo.^19^

### 2.2 Overview of methods informing the design of KONFIDENT

To determine an optimal endpoint to measure the beginning of symptom relief in the phase 3 KONFIDENT trial, we initially engaged patients with HAE on PRO measures in a survey and follow‐up interviews and subsequently conducted post hoc analyses of outcomes from the phase 2 trial to compare the PGI‐C and other PRO measures. Finally, the sample size was determined via a simulation‐based approach using the phase 2 results.

### 2.3 Patient survey and interviews

HAE patient perspectives on PRO measures were collected during a virtual patient advisory board meeting, which was followed by one‐on‐one structured interviews with all patients. Patients were recruited by the US Hereditary Angioedema Association. Feedback was collected on the following outcome measures used in the sebetralstat phase 2 trial: PGI‐C, PGI‐S, and composite VAS for abdominal pain, skin pain, and skin swelling (Figure 2). Feedback was also collected on symptom evolution and the spectrum of symptom relief experienced over the course of HAE attacks. Based on a preliminary discussion during the advisory board meeting, structured feedback was solicited on the clinical meaningfulness of the PGI‐C scale in the context of on‐demand therapy for an HAE attack.

### 2.4 Analyses of agreement between PGI‐C and other PRO measures

Based on patient feedback, the clinical relevance of improvements on the PGI‐C was evaluated through post hoc analyses of its agreement with other outcome measures that were assessed in the phase 2 trial. A cross‐tabulation post hoc analysis was used to evaluate agreement between symptom relief on the PGI‐C (defined as a rating of at least “A Little Better” for two consecutive time points or at least “Better” for one time point within 24 h) and the following outcome measures: (1) improvement on the PGI‐S (defined as at least a one‐level reduction in severity from baseline), (2) improvement on the composite VAS (defined as ≥50% reduction from baseline for three consecutive time points in the composite score of abdominal pain, skin pain, and skin swelling), (3) attack resolution based on the PGI‐S (defined as a rating of “None”), (4) attack resolution based on the VAS (defined as a score of <10 mm for all three components for three consecutive time points), and (5) no use of a conventional on‐demand medication for the attack. All outcome measures were assessed within 24 h of study drug administration. Attacks for which all three VAS components were rated as <10 mm at baseline were excluded from the analysis of attack resolution based on the VAS. The sensitivity of the PGI‐C versus each comparator measure was calculated as the number of true positive outcomes (improvement on the PGI‐C concurrent with improvement/resolution on the comparator outcome) divided by the sum of the number of true positive and false negative outcomes (improvement on the PGI‐C without improvement/resolution on the comparator outcome). Specificity was calculated as the number of true negative outcomes (no improvement on the PGI‐C concurrent with no improvement/resolution on the comparator) divided by the sum of true negative and false positive outcomes (no improvement on the PGI‐C concurrent with improvement/resolution on the comparator outcome). Agreement between the PGI‐C and each comparator was assessed using Cohen's kappa.

### 2.5 Sample size calculation

The sample size for testing the primary endpoint was calculated using a conservative simulation‐based approach to provide at least 90% power for testing each comparison (sebetralstat vs. placebo) with the Feingold and Gillespie score transformation method.^23^ The Bonferroni‐adjusted significance level of 0.025 was obtained by dividing the overall significance level of 0.05 by the number of endpoint family comparisons between each sebetralstat dose level and placebo (0.05 divided by 2). Based on the results of the phase 2 trial, the median time to beginning of symptom relief defined as a PGI‐C rating of at least “A Little Better” for two time points in a row was assumed to be 1.6 h with sebetralstat and 9 h with placebo (ratio of 5.6 with placebo vs. sebetralstat).^19^ The median time to symptom relief was assumed to be identical for both dose levels of sebetralstat to be used in KONFIDENT. The sample size was adjusted to account for patients who do not have three attacks during the treatment period or discontinue the trial (ie, noncompleters), with an approximately 30% noncompleter rate assumed based on the phase 2 trial results.^19^

### 2.6 Ethical considerations

The conduct of the phase 2 trial and the ongoing KONFIDENT phase 3 trial are in accordance with the current Good Clinical Practice Guidelines of the International Council for Harmonization, the Declaration of Helsinki, and all applicable local regulatory requirements. Trial protocols were reviewed and approved by a local ethics committee or institutional review board and competent authorities. Written informed consent or assent was (and will be) obtained from all patients according to local requirements. Patients provided consent for participation in the patient advisory board and one‐on‐one structured follow‐up interviews, and pseudonyms were used to maintain patient confidentiality.

## 3 RESULTS

### 3.1 Patient feedback on PRO measures

Feedback on PRO measures was collected from seven patients with HAE‐C1‐INH (age, 18–74 years; 57% [*n* = 4/7] female; all from the US). Patients had experienced HAE‐C1‐INH symptoms for 8–64 years. In addition to swelling and pain, the most common symptoms of HAE attacks reported by these patients were headache, nausea, issues with bowel movements, and loss of mobility. Icatibant was the most commonly used on‐demand treatment for HAE attacks (*n* = 5/7; 71%), with plasma‐derived C1‐inhibitor (*n* = 1/7; 14%) and recombinant C1‐inhibitor (*n* = 1/7; 14%) also used. Almost all patients (*n* = 5/7; 71%) said that they experienced increased anxiety and stress with the onset of symptoms.

All patients (*n* = 7/7; 100%) indicated that the beginning of symptom relief after treatment of an attack was meaningful to them. Although all (*n* = 7/7; 100%) considered the measured outcomes on the PGI‐C, PGI‐S, and VAS scales meaningful, most (*n* = 5/7; 71%) preferred the PGI‐C to the PGI‐S scale because they felt that the PGI‐C scale increments most appropriately reflected the gradual change experienced by most patients when treating an HAE attack; none preferred the VAS (Table S1). When asked to select the PGI‐C rating (ie, “Much Better,” “Better,” “A Little Better,” “No Change,” “A Little Worse,” “Worse,” or “Much Worse”) that best described overall attack symptoms at the moment they first noticed meaningful improvement following use of an on‐demand treatment, the majority (*n* = 5/7; 71%) selected “A Little Better,” while one patient each selected “Better” and “Much Better.”

### 3.2 Agreement of PGI‐C with other PRO measures in the phase 2 trial

In post hoc analyses of phase 2 data, the PGI‐C rating of at least “A Little Better” was shown to be a highly sensitive measure to identify the beginning of symptom relief (Table 1). The PGI‐C rating of at least “A Little Better” at two consecutive time points demonstrated 97% sensitivity for predicting improvement on both the PGI‐S and composite VAS for abdominal pain, skin pain, and swelling, while the PGI‐C rating of “Better” demonstrated slightly lower sensitivity (92% and 90% for predicting improvements on these measures, respectively). Patient Global Impression of Change ratings of “A Little Better” and “Better” showed fair to substantial agreement with symptom improvement on the PGI‐S (Cohen's kappa of 0.53 and 0.70, respectively) and VAS (Cohen's kappa of 0.67 and 0.78, respectively), with attack resolution on the PGI‐S (Cohen's kappa of 0.39 and 0.59, respectively) and VAS (Cohen's kappa of 0.43 and 0.69, respectively), and with no use of conventional on‐demand treatment (Cohen's kappa of 0.49 and 0.60, respectively). Overall, the PGI‐C rating of “A Little Better” or higher for two consecutive time points showed higher sensitivity but somewhat lower specificity than the rating of “Better”.

**TABLE 1:** Analyses of the Patient Global Impression of Change rating of at least “A Little Better” versus other outcomes.

PGI‐C rating	Comparator	Sensitivity	Specificity	Agreement (Cohen's kappa^a^)
Sensitivity, specificity, and agreement with other outcomes				
“A Little Better”^b^	Improvement on PGI‐S^c^	0.97	0.56	0.53
	Improvement on VAS^d^	0.97	0.67	0.67
	Attack resolution on PGI‐S^e^	0.98	0.46	0.39
	Attack resolution on VAS^f^	0.98	0.46	0.43
	No use of conventional on‐demand treatment	0.86	0.62	0.49
“Better”^g^	Improvement on PGI‐S^c^	0.92	0.78	0.70
	Improvement on VAS^d^	0.90	0.89	0.78
	Attack resolution on PGI‐S^e^	0.96	0.67	0.59
	Attack resolution on VAS^f^	0.96	0.74	0.69
	No use of conventional on‐demand treatment	0.78	0.88	0.60

	Attacks that achieved a PGI‐C rating of at least “A Little Better”^b^	Attacks that did not achieve a PGI‐C rating of at least “A Little Better”^b^
Percentage of attacks that achieved resolution or improvement via other outcomes		
Attacks that achieved symptom improvement on the PGI‐S^c^	70.4% (57/81)	6.3% (2/32)
Attacks that achieved symptom improvement on the VAS^d^	81.5% (66/81)	6.3% (2/32)
Attacks that achieved resolution on the PGI‐S^e^	55.6% (45/81)	3.1% (1/32)
Attacks that achieved complete resolution on the VAS^f^	63.0% (46/73)	4.2% (1/24)
Attacks that required conventional on‐demand therapy	16.0% (13/81)	65.6% (21/32)

Abbreviations: PGI‐C, Patient Global Impression of Change; PGI‐S, Patient Global Impression of Severity; VAS, visual analog scale.

^a^ A Cohen's kappa value of 0.01–0.20 indicates no to slight agreement, 0.21–0.40 indicates fair agreement, 0.41–0.60 indicates moderate agreement, 0.61–0.80 indicates substantial agreement, and 0.81–1.00 indicates almost perfect agreement.

^b^ For two consecutive time points within 24 h.

^c^ Improvement on the PGI‐S was defined as at least a one‐level reduction in severity from baseline.

^d^ Improvement on the VAS was defined as ≥50% reduction in the composite score from baseline.

^e^ Attack resolution on the PGI‐S was defined as a rating of “None”.

^f^ Attack resolution on the VAS was defined as a score of <10 mm for all components for three consecutive time points; attacks with a score of <10 mm for all components at baseline were excluded from analysis.

^g^ For one time point within 24 h.

The PGI‐C rating was also shown to be an early indicator of continued symptom improvement and attack resolution. In attacks for which a PGI‐C rating of at least “A Little Better” (two consecutive time points) was achieved within 4 h, 82.3% (*n* = 51/62) achieved a rating of “Better” (one time point) within 24 h; in those for which a PGI‐C rating of “A Little Better” (two consecutive time points) was achieved within 12 h, 80.0% (*n* = 60/75) achieved a rating of “Better” (one time point) within 24 h. Attacks that achieved a PGI‐C rating of at least “A Little Better” (two consecutive time points) were also more likely to achieve complete attack resolution as indicated by the PGI‐S (55.6% vs. 3.1%) and VAS (63.0% vs. 4.2%) and less likely to require conventional on‐demand treatment (16.0% vs. 65.6%) than attacks that did not achieve a PGI‐C rating of “A Little Better”.

### 3.3 Design of the KONFIDENT phase 3 trial

#### 3.3.1 Endpoints and assessments

**FIGURE 3:**
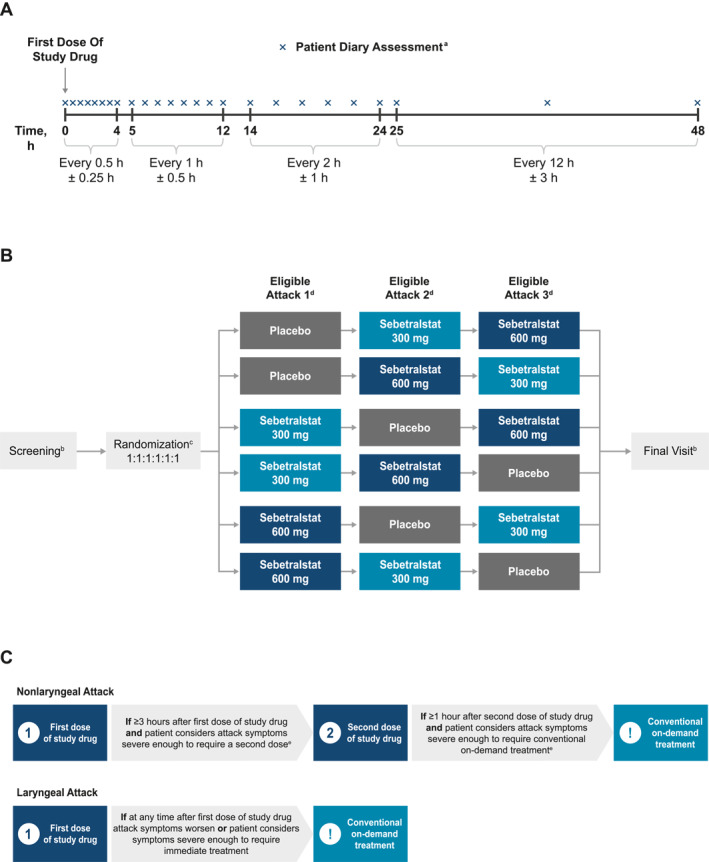
KONFIDENT Study Design. (A) Timing of post‐attack assessments, (B) overall design and treatment arms, and (C) schematic of repeat dosing and conventional on‐demand treatment. ^a^Patients should complete all timed assessments except during sleep but must complete at least the first 4 h of assessments following the first dose of study drug. If a second dose of study drug or treatment with conventional on‐demand treatment is needed, patients will complete diary assessments before each additional dose; the planned post‐dose assessments will then continue through 48 h after the first dose of study drug. ^b^If in‐clinic visits are not possible, home health visits will be permitted; the information captured during home health visits will mirror that captured in an in‐clinic visit. ^c^The randomization visit may occur as an in‐clinic visit or a televisit. ^d^Patients are to contact a study call center after the initial dose of the study drug, prior to a second dose of the study drug, and prior to a dose of conventional on‐demand treatment for each treated attack. ^e^If symptoms progress to airway involvement, patients may be treated with on‐demand treatment at any time.

Based on patient preference as well as its sensitivity and agreement with other outcome measures, time to beginning of symptom relief defined as the PGI‐C rating of at least “A Little Better” for two time points in a row within 12 h from the first dose of study drug was selected as the primary endpoint in the KONFIDENT phase 3 trial (Table 2 and Figure 2). Key secondary endpoints in KONFIDENT are time to first incidence of decrease from baseline (two time points in a row) in PGI‐S score within 12 h of the first dose of study drug and time to attack the resolution defined as “None” on the PGI‐S within 24 h of the first dose of study drug. Other secondary endpoints include proportion of attacks with beginning of symptom relief defined as a PGI‐C rating of at least “A Little Better” for two time points in a row within 4 and 12 h of the first study drug administration, time to PGI‐C rating of at least “Better” for two time points in a row within 12 h of the first study drug administration, time to first incidence of decrease in PGI‐S rating from baseline for two time points in a row within 24 h of the first study drug administration, and time to ≥50% decrease from baseline in the composite VAS score for abdominal pain, skin pain, and skin swelling for three time points in a row (within 12 and 24 h of the first study drug administration; Table 2 and Figure 2). Anxiety will be evaluated as an exploratory endpoint, assessed using a modified version of the General Anxiety–Numeric Rating Scale (Figure 2). Safety will be assessed via the occurrence of adverse events, changes in vital signs, laboratory test results (including hematology, clinical chemistry, and electrolyte panels), and 12‐lead electrocardiogram results. The timing of post‐attack assessments is shown in Figure 3A.

**TABLE 2:** Study endpoints.

Primary endpoint
Time to beginning of symptom relief, defined as a PGI‐C rating of at least “A Little Better” for two time points in a row within 12 h^a^ ^,^ ^b^
Key secondary endpoints
Time to first decrease from baseline in PGI‐S for two time points in a row within 12 h^a^ ^,^ ^b^
Time to attack resolution, defined as “None” on the PGI‐S within 24 h^a^ ^,^ ^b^
Secondary endpoints
Proportion of attacks with symptom relief, defined as a PGI‐C rating of at least “A Little Better” for two time points in a row within 4 and 12 h^a^
Time to PGI‐C rating of at least “Better” for two time points in a row within 12 h^a^
Time to first decrease from baseline in PGI‐S for two time points in a row within 24 h^a^
Time to ≥50% decrease from baseline for three time points in a row in composite VAS within 12 and 24 h^a^
Exploratory endpoint
Cumulative GA‐NRS rating expressed as area under the curve over 12 and 24 h^a^

Abbreviations: GA‐NRS, General Anxiety–Numeric Rating Scale; PGI‐C, Patient Global Impression of Change; PGI‐S, Patient Global Impression of Severity; VAS, visual analog scale.

^a^ From the first dose of study drug.

^b^ Primary and key secondary endpoints will be tested according to the fixed‐sequence closed testing procedure.

The primary and key secondary endpoints confirmatory efficacy analysis will be adjusted for multiplicity; other secondary and exploratory endpoints will not be adjusted for multiplicity. A hierarchical, fixed‐sequence, closed‐testing procedure with a loop‐back feature to allow two‐way alpha passing will be used to test the primary and key secondary endpoints, separately for each dose comparison to placebo. Initially, the first key secondary endpoint, time to first incidence of decrease from baseline in PGI‐S for two time points in a row within 12 h of the first dose of study drug, will be tested only if the test on the primary endpoint reaches statistical significance (alpha of 0.025). The second key secondary endpoint, time to attack resolution defined as “None” on the PGI‐S within 24 h of the first dose of the study drug, will be tested only if the test on the first key secondary endpoint reaches a significance level of 0.025. The initial testing within a dose level will be stopped if the previous test in the sequence does not reach a significance level of 0.025. If the primary and key secondary endpoint hypotheses within one of the pairwise comparisons are not all rejected at the 0.025 alpha level, but the hypotheses for the other pairwise comparison are all rejected, then the unused alpha from the rejected hypotheses can be directed to loop back to the unrejected hypotheses, which is then retested at the alpha level of 0.05. If the testing procedure is stopped eventually, the remaining endpoints in the testing sequence will be considered exploratory.

#### 3.3.2 Eligibility criteria

The KONFIDENT trial will include patients in North American, European, and Asia‐Pacific countries. Enrollment began in March 2022 in the US, and target enrollment was achieved as of July 7, 2023. Key inclusion and exclusion criteria for the KONFIDENT trial are shown in Table 3. This trial will include adolescents and adults receiving prophylactic or on‐demand treatment regimens who meet the minimum attack frequency requirement at baseline. These criteria expand the eligible patient population compared with the earlier phase 2 trial, which was limited to adults who were not on prophylactic treatment.^19^ Inclusion of adolescents was supported by a population pharmacokinetic model that predicted that sebetralstat exposure following a single 600‐mg dose would be similar for adolescents and adults.^24^

**TABLE 3:** Key eligibility criteria.

Key inclusion criteria
Age ≥12 years
Confirmed diagnosis of HAE type I or II (ie, HAE‐C1‐INH)
Access to and ability to use conventional on‐demand treatment for HAE attacks
At least two documented HAE attacks within 3 months prior to randomization
On a stable dose and regimen for ≥3 months prior to screening if receiving long‐term prophylactic treatment with IV or SC plasma‐derived C1‐INH, lanadelumab, and/or berotralstat^a^
Last dose of attenuated androgens ≥28 days prior to randomization
Key exclusion criteria
Any concomitant diagnosis of another form of chronic angioedema^b^
Clinically significant history of poor response to BR2 blocker, C1‐INH therapy, or plasma kallikrein inhibitor therapy for the management of HAE‐C1‐INH
Use of protocol‐defined prohibited medications^c^
Any clinically significant comorbidity or systemic dysfunction
Participation in any gene therapy treatment or trial for HAE‐C1‐INH

Abbreviations: BR2, bradykinin receptor 2; C1‐INH, C1‐inhibitor; HAE, hereditary angioedema; HAE‐C1‐INH, hereditary angioedema with C1‐inhibitor deficiency; IV, intravenous; SC, subcutaneous.

^a^ Patients must be willing to remain on a stable dose and regimen for the duration of the trial.

^b^ Includes acquired C1‐INH deficiency, HAE with normal C1‐INH, idiopathic angioedema, and angioedema associated with urticaria.

^c^ Includes use of angiotensin‐converting enzyme inhibitors after the screening visit or within 7 days prior to randomization, use of any estrogen‐containing medication with systemic absorption after the screening visit or within 7 days prior to randomization, and use of strong cytochrome P450 3A4 inhibitors or inducers during participation in the trial starting at the screening visit.

Eligible HAE attacks must meet the following criteria: (1) the attack is not a severe laryngeal attack as defined by patient‐reported PGI‐S, (2) the patient can identify the start time of the attack, (3) ≥48 h have elapsed since conventional on‐demand treatment or study drug was used to treat an attack, and (4) the patient can complete at least the first 4 h of diary assessments following the first administration of study drug.

#### 3.3.3 Treatment and study design

Patients will be randomized to one of six treatment sequences in a three‐way crossover design, in which three eligible HAE attacks will be treated with placebo, sebetralstat 300 mg, or sebetralstat 600 mg (Figure 3B). After attack recognition, patients self‐administer a single dose of study drug; each eligible attack may be treated with up to two doses of the assigned study drug (Figure 3C). For attacks not involving the larynx, a second dose of the study drug may be taken at least 3 h after the first dose if symptoms are worsening or considered severe enough by the patient. Conventional on‐demand treatment may be taken ≥1 h after the second dose if symptoms continue to worsen or are still considered severe enough by the patient, or at any time if symptoms progress to airway involvement. For eligible laryngeal attacks (ie, those assessed by patients as mild or moderate severity), patients will administer a single dose of study drug and contact their study physician shortly thereafter for further evaluation (eg, confirmation of attack severity, assessment of need for additional medical intervention). If laryngeal symptoms worsen or are considered severe enough by the patient after the first dose of study drug, conventional on‐demand treatment will be administered immediately rather than a second dose of study drug. For all eligible attacks, patients will contact the study call center for guidance regarding next steps in the trial and the trial diary as follows: (1) following the initial dose of study drug, (2) prior to the second dose of study drug, and (3) prior to a dose of conventional on‐demand treatment. HAE attacks that do not meet eligibility criteria may be treated with conventional on‐demand treatment (including plasma‐derived C1‐inhibitor, recombinant human C1‐esterase inhibitor, icatibant, and ecallantide) per the patient's usual treatment regimen. A 48‐h washout period is required prior to subsequent dosing with the study drug.

#### 3.3.4 Sample size

A sample size of 84 patients completing treatment of three HAE attacks (approximately 14 per treatment sequence) was estimated to provide at least 90% power for testing each pairwise comparison for the primary endpoint in the KONFIDENT trial. Accounting for noncompleters, the target enrollment was approximately 114 patients to ensure that approximately 84 patients, including approximately 12 adolescents, completed the trial.

## 4 DISCUSSION

To identify an optimal outcome measure for the assessment of efficacy for the on‐demand treatment of HAE attacks, we used a multipronged approach that included perspectives of patients with HAE and post hoc analyses of the phase 2 endpoints of improvements assessed using PRO instruments (PGI‐C, PGI‐S, and VAS). Feedback from a patient advisory board initially identified the PGI‐C as a preferred PRO for assessing symptom relief in HAE, and follow‐up structured interviews highlighted that the rating of “A Little Better” on the PGI‐C best represented the earliest signs of symptom relief for most patients. Subsequent post hoc analyses of phase 2 data demonstrated that the PGI‐C rating of at least “A Little Better” for two time points in a row is a sensitive, clinically meaningful measure of the efficacy of on‐demand treatments for HAE attacks. Taken together, these results provide a strong rationale for the selection of time to the beginning of symptom relief assessed via the PGI‐C rating of at least “A Little Better” at two time points in a row within 12 h as the primary endpoint in the phase 3 KONFIDENT trial.

Using a randomized, event‐driven, six‐sequence, three‐way crossover design, KONFIDENT was designed to confirm the efficacy and safety of sebetralstat, an investigational oral therapy for on‐demand treatment of HAE‐C1‐INH. This trial will augment the existing evidence from phase 1 and 2 trials, which demonstrated that sebetralstat had favorable pharmacokinetics and pharmacodynamics, was generally safe and well tolerated, and led to rapid symptom relief in patients with HAE‐C1‐INH.^19^, ^25^, ^26^ Due to novel additions to the phase 2 trial design, KONFIDENT will provide a broader understanding of the efficacy and safety of sebetralstat for the on‐demand treatment of attacks in patients with HAE‐C1‐INH. Compared with the phase 2 trial,^19^ KONFIDENT's eligibility criteria have been broadened to include adolescents, patients who have attacks while receiving nearly all forms of approved long‐term prophylactic treatment, and attacks in all anatomical locations and levels of severity, with the exception, for ethical reasons, of laryngeal attacks considered severe at onset by patients. This study will also allow for the use of a second dose of blinded study drug to enable further treatment of attacks with persisting or worsening symptoms.

KONFIDENT is the first randomized controlled trial to evaluate the efficacy of on‐demand treatment for HAE attacks occurring in patients receiving stable, long‐term prophylactic therapy. It is also the first on‐demand trial that does not include attack severity as an eligibility criterion for enrollment. Prior trials for earlier on‐demand medications required a minimum level of severity, typically at least moderate,^27^, ^28^, ^29^ which may have led to treatment delays while patients awaited adequate worsening of attacks to be eligible for treatment. Additionally, KONFIDENT is designed to enable earlier treatment than the phase 2 trial of sebetralstat, in which patients had to contact their study physician to confirm attack eligibility prior to self‐administering sebetralstat.^19^ Thus, it is expected that the treatment conditions in KONFIDENT will be closer to those in the real world and align more closely with reported patient experience. Finally, KONFIDENT is the first on‐demand study using the General Anxiety–Numeric Rating Scale to evaluate changes in anxiety during treatment of HAE attacks. Understanding the anxiety associated with HAE‐C1‐INH attacks may help contribute to overall patient well‐being.

As an oral, on‐demand therapy, sebetralstat has the potential to enable early treatment of HAE‐C1‐INH attacks. Treating all attacks as early as possible, regardless of severity, is important to reduce the overall duration and severity of attacks, decrease their impact on daily activities, and improve patient quality of life.^3^, ^4^, ^30^ Further, by preventing attack progression, early intervention is likely to lower the overall cost and burden of HAE, including decreasing the number of emergency department visits and hospitalizations and loss of work productivity,^31^ supporting the overall value of early treatment of HAE attacks.

In conclusion, the PGI‐C rating of at least “A Little Better” for two time points in a row is a clinically meaningful, patient‐preferred efficacy outcome measure to detect the earliest signs of symptom relief following on‐demand treatment for HAE attacks and is predictive of other efficacy measures, including attack resolution. Time to beginning of symptom relief assessed via this PGI‐C rating has therefore been chosen as the primary efficacy endpoint in the KONFIDENT phase 3 trial of sebetralstat. Sebetralstat has the potential to become the first oral therapy for on‐demand treatment of HAE‐C1‐INH, thereby addressing an unmet need and shifting the HAE‐C1‐INH treatment landscape.

## AUTHOR CONTRIBUTIONS

**Danny M. Cohn**: Conceptualization (equal); formal analysis (equal); methodology (equal); project administration (equal); visualization (equal); writing—review and editing (equal). **Emel Aygören‐Pürsün**: Writing—review and editing (equal); **Jonathan A. Bernstein:** Investigation (equal); writing—review and editing (equal). **Henriette Farkas**: Writing—review and editing (equal). **William R. Lumry**: Investigation (equal); supervision (equal); writing—review and editing (equal). **Marcus Maurer**: Writing—review and editing (equal). **Andrea Zanichelli**: Writing—review and editing (equal). **Matthew Iverson**: Methodology (equal); project administration (equal); supervision (equal); writing—review and editing (equal). **James Hao**: Formal analysis (equal); writing—review and editing (equal). **Michael D. Smith**: Conceptualization (equal); formal analysis (equal); methodology (equal); project administration (equal); supervision (equal); writing—review and editing (equal). **Christopher M. Yea**: Writing—review and editing (equal); conceptualization (equal); methodology (equal); supervision (equal). **Paul K. Audhya**: Conceptualization (equal); data curation (lead); formal analysis (equal); investigation (equal); methodology (equal); supervision (equal); validation (equal); visualization (equal); writing—original draft (lead); writing—review and editing (equal). **Marc A. Riedl**: Conceptualization (equal); investigation (equal); methodology (equal); supervision (equal); validation (equal); writing—review and editing (equal).

## CONFLICT OF INTEREST STATEMENT

Danny M. Cohn has received speaker, consultancy, and/or advisory board fees from BioCryst, CSL Behring, Ionis Pharmaceuticals, KalVista, Pharvaris, and Takeda; Danny M. Cohn is also a member of the HAE International Medical Advisory Panel. Emel Aygören‐Pürsün has received grants, speaker fees, consultancy fees, travel support, advisory board fees, and/or honoraria for manuscript writing and educational events from BioCryst, BioMarin, Centogene, CSL Behring, KalVista, Pharming, Pharvaris, and Shire/Takeda. Jonathan A. Bernstein has received grants and/or speaker/consultancy fees from and served on advisory boards for Astria, BioCryst, BioMarin, CSL Behring, Cycle Pharmaceuticals, Incyte, KalVista, Ono, Pharming, Pharvaris, and Shire/Takeda; Jonathan A. Bernstein is also a member of the medical advisory board for the US HAE Association. Henriette Farkas has received research grants from CSL Behring, Pharming, and Takeda; and has served as an advisor and speaker for these companies and for Astria, BioCryst, KalVista, and Ono. William R. Lumry has received grants and/or speaker/consultancy fees from and/or served on advisory boards for Astria, BioCryst, BioMarin, CSL Behring, Fresenius Kabi, Intellia, Ionis, KalVista, Pharming, and Takeda; William R. Lumry is also a member of the medical advisory board for the US HAE Association. Marcus Maurer is or recently was a speaker and/or advisor for and/or has received research funding from Arcensus, Astria, BioCryst, Centogene, CSL Behring, KalVista, Pharming, Pharvaris, and Takeda. Andrea Zanichelli has received speaker/consultancy fees from BioCryst, CSL Behring, KalVista Pharmaceuticals, Pharming, and Takeda. Matthew Iverson, James Hao, Michael D. Smith, Christopher M. Yea, and Paul K. Audhya are employees of KalVista Pharmaceuticals. Marc A. Riedl has received research funding from BioCryst, BioMarin, CSL Behring, Ionis, KalVista, Pharvaris, and Takeda, and advisor and/or speaker fees from Astria, Biocryst, Biomarin, CSL Behring, Cycle, Intellia, Ipsen, KalVista, Ono, Pfizer, Pharming, Pharvaris, RegenexBio, Sanofi‐Regeneron, Spark, and Takeda.

## TRIAL REGISTRATION

NCT05259917, NCT04208412.

## Supporting information

Supporting Information S1

## Data Availability

Reasonable requests from qualified scientific and medical researchers for data sharing will be considered; please email your inquiries to: medinfo@kalvista.com.

## References

[1] Longhurst H , Cicardi M . Hereditary angio‐oedema. Lancet. 2012;379(9814):474‐481. 10.1016/s0140-6736(11)60935-5 22305226

[2] Aygören‐Pürsün E , Magerl M , Maetzel A , Maurer M . Epidemiology of bradykinin‐mediated angioedema: a systematic investigation of epidemiological studies. Orphanet J Rare Dis. 2018;13(1):73. 10.1186/s13023-018-0815-5 29728119 PMC5935942

[3] Maurer M , Magerl M , Betschel S , et al. The international WAO/EAACI guideline for the management of hereditary angioedema—the 2021 revision and update. Allergy. 2022;77(7):1961‐1990. 10.1016/j.waojou.2022.100627 35006617

[4] Niccola S , Rolla G , Brussino L . Breakthroughs in hereditary angioedema management: a systematic review of approved drugs and those under research. Drugs Context. 2019;8:212605. 31645881 10.7573/dic.212605 PMC6788388

[5] Orladeyo (Berotralstat) Precribing Information. BioCryst Pharmaceuticals, Inc. 2022.

[6] Takhzyro (Lanadelumab‐flyo) Prescribing Information. Takeda Pharmaceuticals USA, Inc. 2022.

[7] Haegarda (C1 Esterase Inhibitor Subcutaneous [human]) Prescribing Information. CSL Behring LLC. 2022.

[8] Cinryze (C1 Esterase Inhibitor [human]) Prescribing Information. Takeda Pharmaceuticals USA, Inc. 2021.

[9] Berinert (C1 Esterase Inhibitor [human]) Prescribing Information. CSL Behring LLC. 2021.

[10] Ruconest (C1 Esterase Inhibitor [recombinant]) Prescribing Information. Pharming Healthcare Inc. 2020.

[11] Firazyr (Icatibant) Prescribing Information. Takeda Pharmaceuticals America, Inc. 2021.

[12] Kalbitor (Ecallantide) Prescribing Information. Takeda Pharmaceutical Company Limited. 2020.

[13] Aberer W , Maurer M , Bouillet L , et al. Breakthrough attacks in patients with hereditary angioedema receiving long‐term prophylaxis are responsive to Icatibant: findings from the Icatibant Outcome Survey. Allergy Asthma Clin Immunol. 2017;13(1):31. 10.1186/s13223-017-0203-z 28690642 PMC5497380

[14] Hernández Fernandez de Rojas D , Ibañez E , Longhurst H , et al. Treatment of HAE attacks in the Icatibant Outcome Survey: an analysis of icatibant self‐administration versus administration by health care professionals. Int Arch Allergy Immunol. 2015;167(1):21‐28. 10.1159/000430864 26112099

[15] Zanichelli A , Azin G , Cristina F , Vacchini R , Caballero T . Safety, effectiveness, and impact on quality of life of self‐administration with plasma‐derived nanofiltered C1 inhibitor (Berinert®) in patients with hereditary angioedema: the SABHA study. Orphanet J Rare Dis. 2018;13(1):51. 10.1186/s13023-018-0797-3 29631595 PMC5891972

[16] US Food and Drug Administration Center for Biologics Evaluation and Research . The Voice of the Patient: Hereditary Angioedema; 2018. Accessed 25 January 2023. https://www.fda.gov/media/113509/download

[17] Dempster J . Practicalities of a reduced volume formulation of a C1‐INH concentrate for the treatment of hereditary angioedema: real‐life experience. Allergy Asthma Clin Immunol. 2018;14(1):44. 10.1186/s13223-018-0267-4 30386384 PMC6201499

[18] Tuong L.‐A , Olivieri K , Craig T . Barriers to self‐administered therapy for hereditary angioedema. Allergy Asthma Proc. 2014;35(3):250‐254. 10.2500/aap.2014.35.3753 24801468

[19] Aygören‐Pürsün E , Zanichelli A , Cohn D , et al. Investigational oral plasma kallikrein inhibitor sebetralstat for on‐demand treatment of hereditary angioedema. Lancet. 2023;401(10375):458‐469. 10.1016/s0140-6736(22)02406-0 36774155

[20] Fijen L , Petersen R , Cohn S . Outcome measures in randomized controlled studies of acute therapy for hereditary angioedema: a systematic review. Allergy. 2022;77(7):2222‐2224. 10.1111/all.15244 35122275 PMC9305446

[21] Maurer M , Aberer W , Bouillet L , et al. Hereditary angioedema attacks resolve faster and are shorter after early icatibant treatment. PLOS One. 2013;8(2):e53773. 10.1371/journal.pone.0053773 23390491 PMC3563637

[22] Zanichelli A . Fast Improvement of HAE Attacks with the Oral On‐Demand Plasma Kallikrein Inhibitor KVD900: An Analysis of the Pharmacokinetic and Pharmacodynamic Profile of KVD900 and Attack Symptom Severity during a Double‐Blind, Randomized Phase 2 Cross‐Over Trial in Patients with HAE Type I and II. Presented at: 12th C1‐Inhibitor Deficiency and Angioedema Workshop; 2021.

[23] Feingold M , Gillepsie B . Cross‐over trials with censored data. Stat Med. 1996;15(10):953‐967. 10.1002/(sici)1097-0258(19960530)15:10<953::aid-sim213>3.0.co;2-m 8783435

[24] Smith M , Rodgers T , Sharman J , et al. Population Pharmacokinetic Analysis of KVD900 in Healthy Adult Volunteers and Patients with Hereditary Angioedema Predicts Similar Exposure in Adolescent Patients. Presented at: EAACI Hybrid Congress 2022; 2022. Abstract 001360.

[25] Duckworth E , Murugesan N , Li L , et al. Pharmacological suppression of the kallikrein kinin system with KVD900: an orally available plasma kallikrein inhibitor for the on‐demand treatment of hereditary angioedema. Clin Exp Allergy. 2022;52(9):1059‐1070. 10.1111/cea.14122 35278245 PMC9544254

[26] Maetzel A , Smith M , Duckworth E , et al. KVD900, an oral on‐demand treatment for hereditary angioedema: phase 1 study results. J Allergy Clin Immunol. 2022;149(6):2034‐2042. 10.1016/j.jaci.2021.10.038 35086692

[27] ClinicalTrials.gov . A Study of Icatibant in Patients with Acute Attacks of Hereditary Angioedema (FAST‐3). Accessed 18 November 2022. https://clinicaltrials.gov/ct2/show/NCT00912093

[28] ClinicalTrials.gov . Efficacy Study of DX‐88 (Ecallantide) to Treat Acute Attacks of Hereditary Angioedema (HAE). Accessed 18 November 2022. https://clinicaltrials.gov/ct2/show/NCT00457015

[29] ClinicalTrials.gov . Recombinant Human C1 Inhibitor for the Treatment of Acute Attacks in Patients with Hereditary Angioedema. Accessed 18 November 2022. https://clinicaltrials.gov/ct2/show/NCT00262301

[30] Radojicic C , Manning M , Guilarte M , et al. Patient Perspectives on Early Use of On‐Demand Treatment for Hereditary Angioedema (HAE) Attacks to Reduce Severity and Duration. Presented at: American Academy of Allergy, Asthma, and Immunology; 2023.

[31] Lumry WR . Hereditary angioedema: the economics of treatment of an orphan disease. Front Med. 2018;5(22). 10.3389/fmed.2018.00022 PMC5820358 29503818

