# Supplementary material for: Evaluation of patient‐reported outcome measures for on‐demand treatment of hereditary angioedema attacks and design of KONFIDENT, a phase 3 trial of sebetralstat
Source: Clin Transl Allergy. 2023 Sep 4;13(9):e12288. doi: 10.1002/clt2.12288 (PMC10476273; doi:10.1002/clt2.12288)
Supplement: Supplementary file 1 — Supporting Information S1 [file CLT2-13-e12288-s001.docx]

# SUPPORTING INFORMATION

**Table S1. Summary of Patient Feedback on PROs**

|  | **Patients, n/N (%)** |
| --- | --- |
| **Preference for PRO measures** |  |
| Preferred the PGI-C to the PGI-S | 5/7 (71) |
| Did not express any strong opinion about the scale | 2/7 (29) |
| Preferred the VAS | 0/7 |
| **Description of overall HAE attack symptoms when improvement was first noticed after treatment^a^** |  |
| Much Better | 1/7 (14) |
| Better | 1/7 (14) |
| A Little Better | 5/7 (71) |
| Same | 0/7 |
| A Little Worse | 0/7 |
| Worse | 0/7 |
| Much Worse | 0/7 |

HAE, hereditary angioedema; PGI-C, Patient Global Impression of Change; PGI-S, Patient Global Impression of Severity; PRO, patient-reported outcome; VAS, visual analog scale.

^a^ Question: “At the moment when you first notice the medicine is beginning to work, how would you describe your overall HAE attack symptoms?”
